# Supplementary material for: Evidence supported by Mendelian randomization: impact on inflammatory factors in knee osteoarthritis
Source: Front Med (Lausanne). 2024 May 28;11:1382836. doi: 10.3389/fmed.2024.1382836 (PMC11165061; doi:10.3389/fmed.2024.1382836)
Supplement: Supplementary file 1 [file Data_Sheet_1.ZIP › data/GCST90274838_5e6/838.forest.pdf]

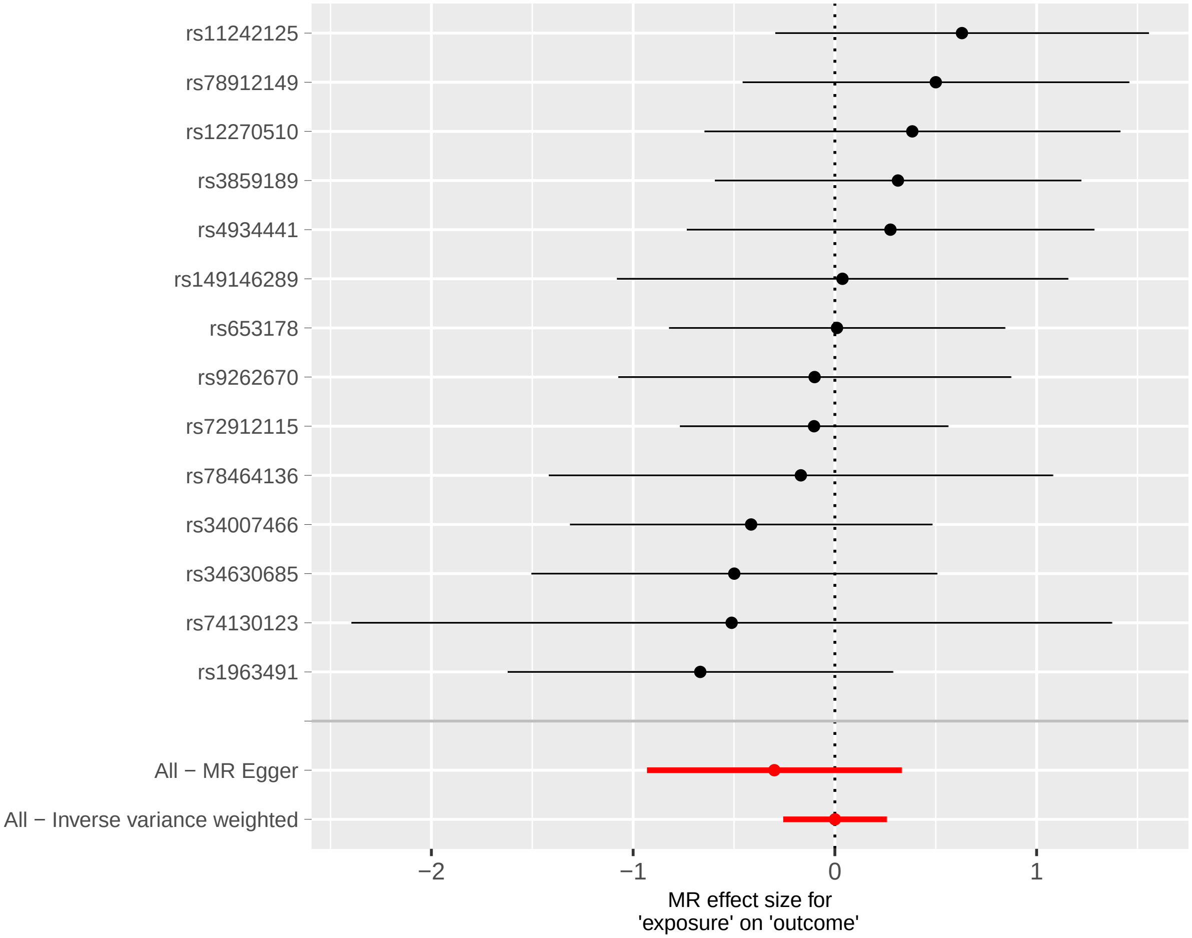
rs11242125
rs78912149
rs12270510
rs3859189
rs4934441
rs149146289
rs653178
rs9262670
rs72912115
rs78464136
rs34007466
rs34630685
rs74130123
rs1963491
All − MR Egger
All − Inverse variance weighted
−2
−1
0
1
MR effect size for
'exposure' on 'outcome'
